# Supplementary material for: Tetracistronic minigenomes elucidate a functional promoter for Ghana virus and unveils Cedar virus replicase promiscuity for all henipaviruses
Source: J Virol. 2024 Sep 30;98(10):e00806-24. doi: 10.1128/jvi.00806-24 (PMC11495047; doi:10.1128/jvi.00806-24)
Supplement: Supplemental figures — Figures S1 through S8. [file jvi.00806-24-s0001.docx]

**
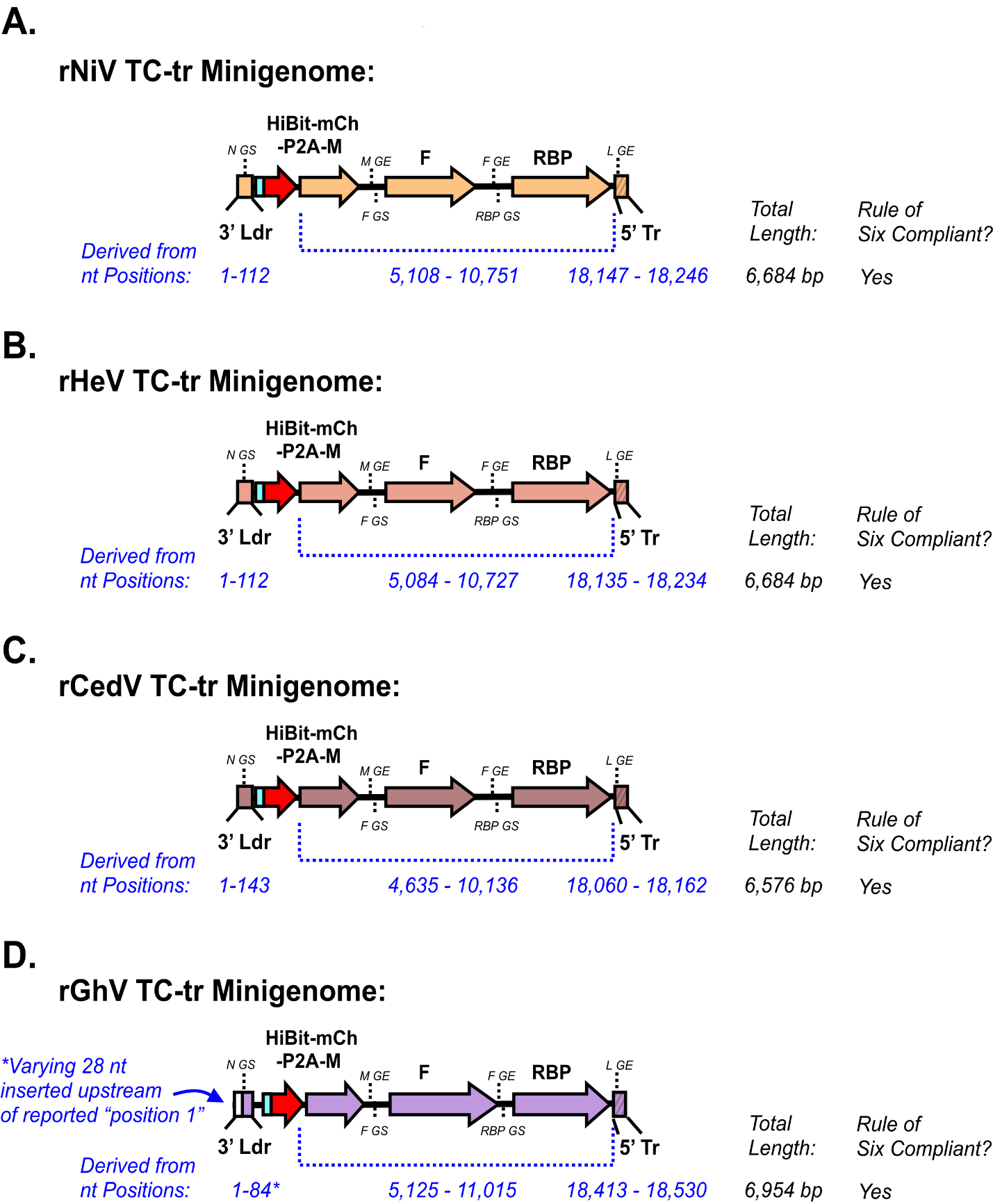
**

**Supplementary Figure 1. Schematic representation of rHNV TC-tr minigenome construct designs.** Tetracistronic henipavirus minigenomes were designed for **(A)** Nipah virus, **(B)** Hendra virus, **(C)** Cedar virus, **(D)** and Ghana virus. All constructs encode the respective reported 3’ Ldr sequence through the start codon of the N gene, the viral sequence spanning the start codon of the M gene through the stop codon of the RBP gene, and at least 100 nucleotides derived from the 5’ Tr sequence. All constructs were designed to be compliant with the rule of six; if required, additional stop codons were added after the RBP gene to fulfill this rule. Each panel details which respective regions of the viral genome were assembled together, with nucleotide positions corresponding to the accession sequence: For NiV, derived sequences are from strain UMMC1 (GenBank AY029767.1); for HeV, derived sequences are from HeV/Australia/1994/Horse18 (MN062017.1); for CedV, derived sequences are from strain CG1a (GenBank JQ001776.1); and for GhV, derived sequences are from strain Eid_hel/GH-M74a/GHA/2009 (NCBI Reference Sequence NC_025256.1). Note that for GhV (**D**), an additional 28 nucleotides are inserted upstream of the reported 3’Ldr sequence for all constructs.


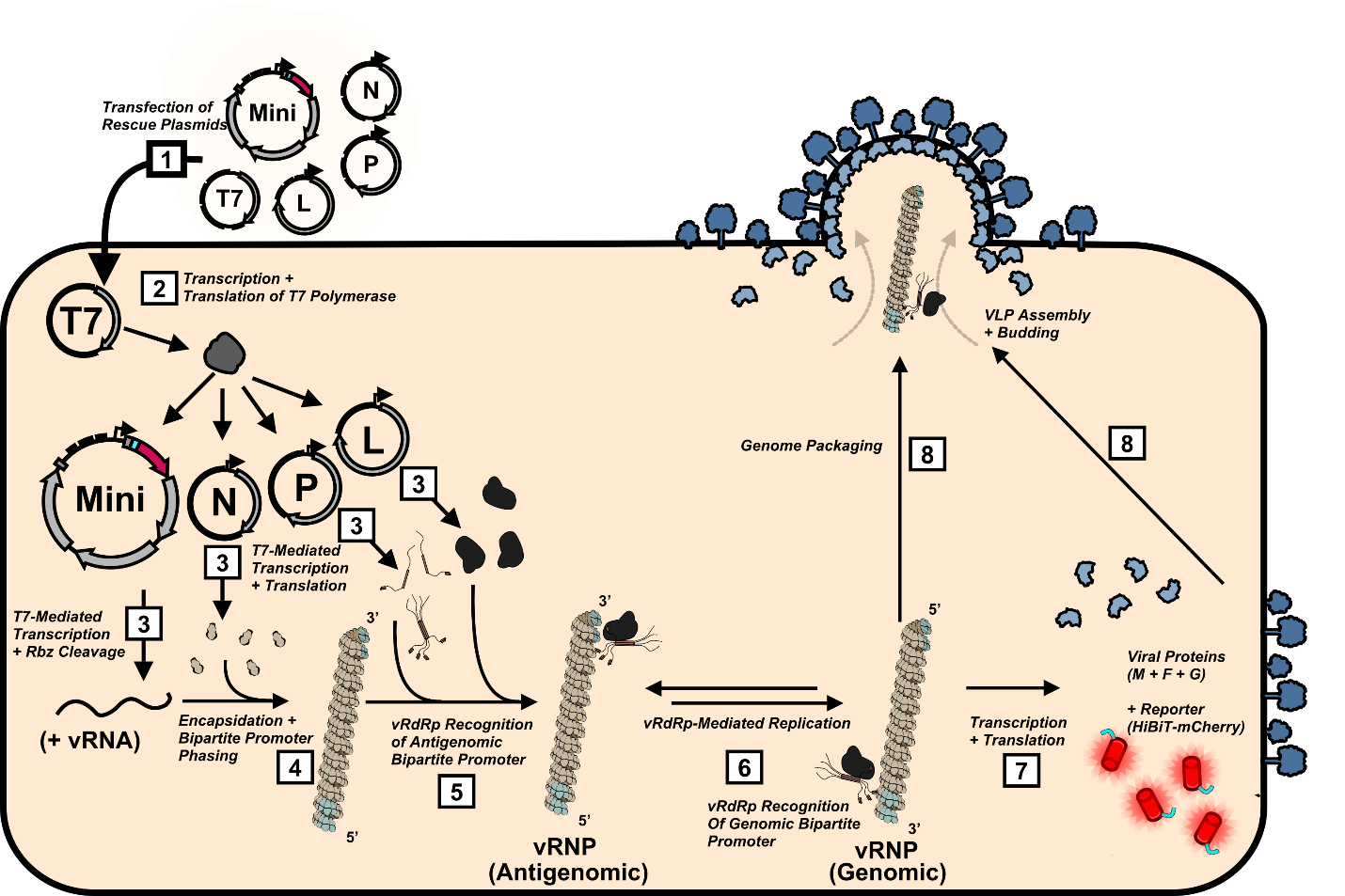


**Supplementary Figure 2. Schematic detailing TC-tr minigenome rescue.** For minigenome rescue to occur, plasmids encoding the rHNV TC-tr minigenome, T7-HNV-N, T7-HNV-P, T7-HNV-L, and codon-optimized T7 polymerase must be cotransfected into cells (1). Following co-transfection, T7 polymerase is transcribed and translated (2). T7 polymerase drives transcription of HNV-N, -P, -L, and the rHNV TC-tr minigenome in the cellular cytoplasm. As the rHNV TC-tr minigenomic RNA is transcribed, the ribozyme elements at the terminal ends fold and cleave, resulting in antigenomic vRNA without exogenous sequence on the ends (3). HNV-N binds to and oligomerizes along the length of the minigenomic vRNA, resulting in the three-dimensional phasing of the antigenomic bipartite promoter onto the same surface of the vRNP (4). Upon proper phasing of the antigenomic bipartite promoter, HNV-P may recruit HNV-L to the vRNP, forming the replicase (5). HNV-L may then recognize the antigenomic bipartite promoter, resulting in vRdRp dependent replication and genomic minigenomic vRNA. Proper phasing of the genomic bipartite promoter results in recognition by the vRdRp, resulting in further rounds of replication (6) and transcription (7) of genes encoded by the minigenomic vRNA. Expression of HiBiT-mCherry provides a visual and quantitative readout for vRdRp activity, and expression of HNV-M, -F-, and -RBP can result in packaging of the minigenomic vRNP into TC-tr VLPs (8).


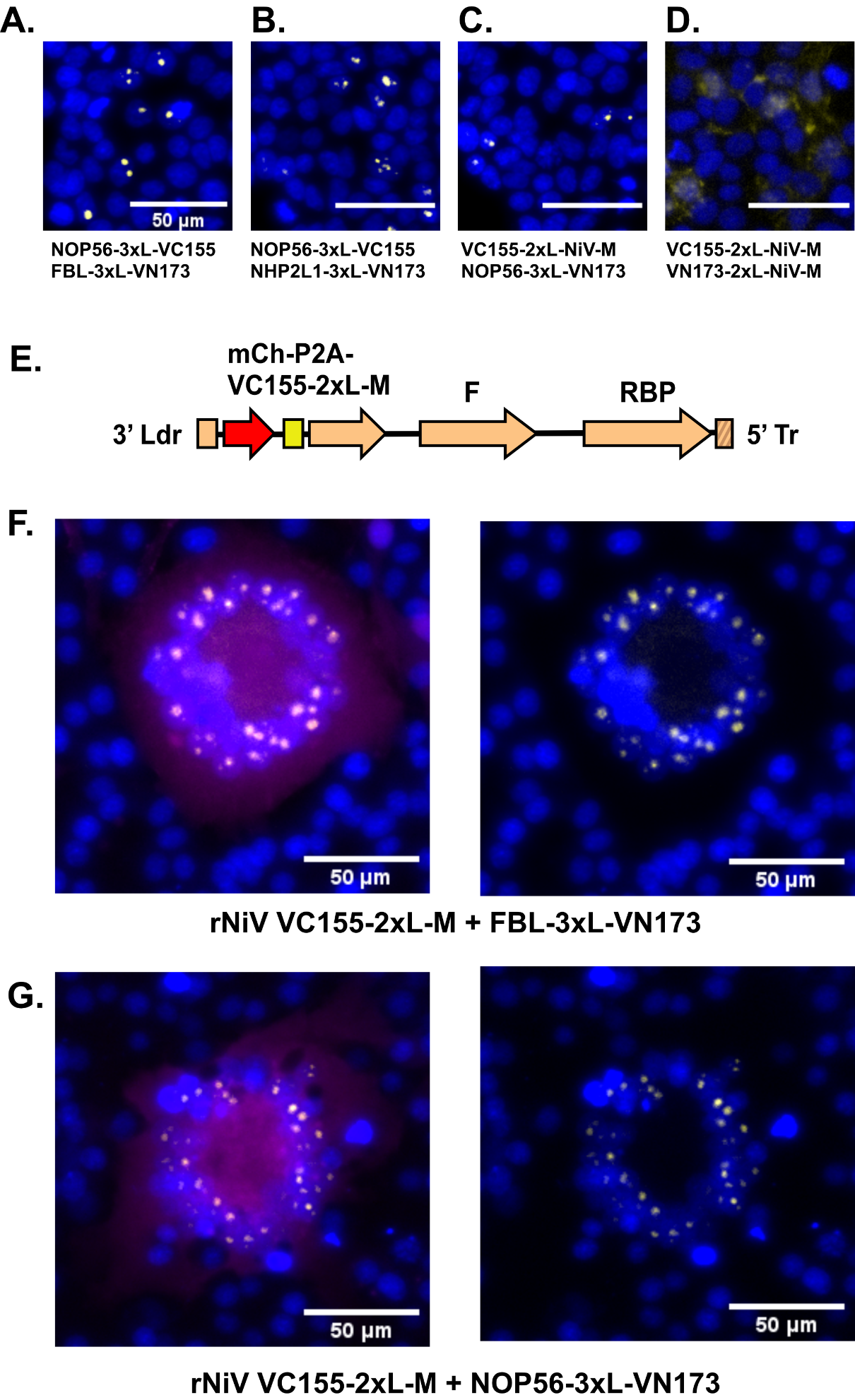


**Supplementary Figure 3. Validation of BiFC constructs.** Co-transfection of HEK-293T cells with (A) NOP56-3xL-VC155 and FBL-3xL-VN173, or (B) NOP56-3xL-VC155 and NHP2L1-3xL-VN173 results in BiFC signal exclusively in nucleolar punctae. Further, co-transfection of HEK-293T cells with (C) VC155-2xL-NiV-M with NOP56-3xL-VN173, but not (D) VC155-2xL-NiV-M with VN173-2xL-NiV-M, yields BiFC in nucleoli. (E) Design of a rNiV TC-tr minigenome encoding VC155 tethered to the N-terminus of NiV-M, with the two separated by a 2x GGGGS linker. Rescue of the rNiV TC-tr minigenome encoding VC155-2xL-NiV-M in BSRT7 cells co-transfected with (F) FBL-3xL-VN173 or (G) NOP56-3xL-VN173 yields mCherry-positive syncytia with BiFC localized in nucleolar punctae. Both (F) and (G) show panels with mCherry signal (left) and without (right) for better visualization of nuclei and BiFC.


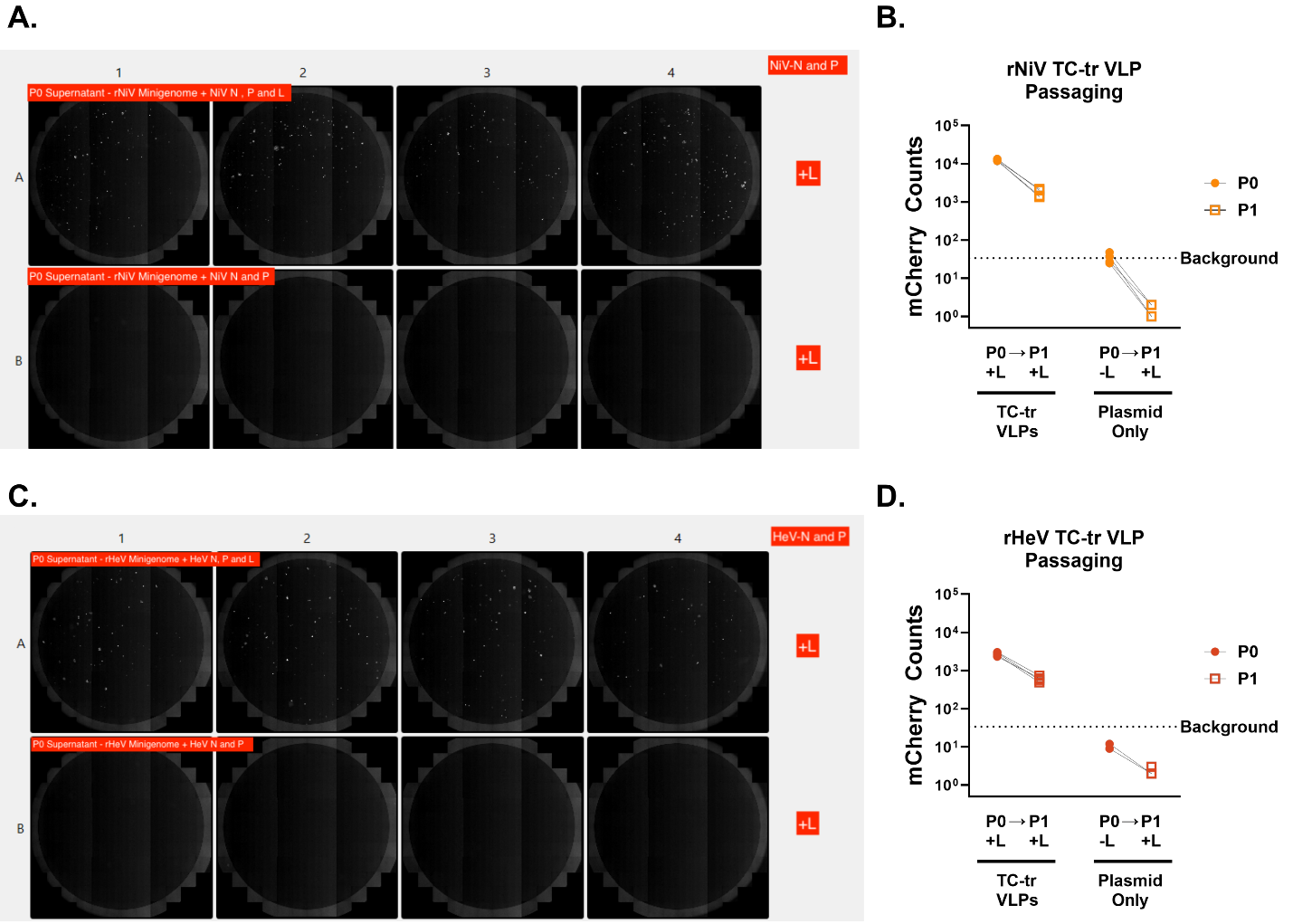


**Supplementary Figure 4. Passaging of rHNV minigenomes is TC-trVLP dependent.** The rNiV and rHeV TC-tr minigenomes, respectively, were transfected into cells as described, either in the presence or absence of respective HNV-L for the P0 rescue of TC-trVLPs. P0 rescue with HNV-L will result in TC-trVLPs while P0 transfection in the absence of HNV-L will not, providing a control to measure if residual antigenomic plasmid cDNA in the rescue supernatant is capable of driving infection events during passaging. Rescue wells were imaged and mCherry events were quantified for P0. Supernatant from P0 was then used to infect BSRT7 cells pre-transfected with HNV-N/-P/-L and codon optimized T7 polymerase for passage 1 (P1). The P1 wells were imaged at 96HPI for (A) rNiV TC-tr systems and mCherry counts were (B) quantified and plotted as compared to the original P0 rescue wells. Likewise, P1 wells were imaged at 96HPI for (C) rHeV TC-tr systems and mCherry counts were (D) quantified and plotted as compared to the original rHeV TC-tr minigenome P0 rescue wells.


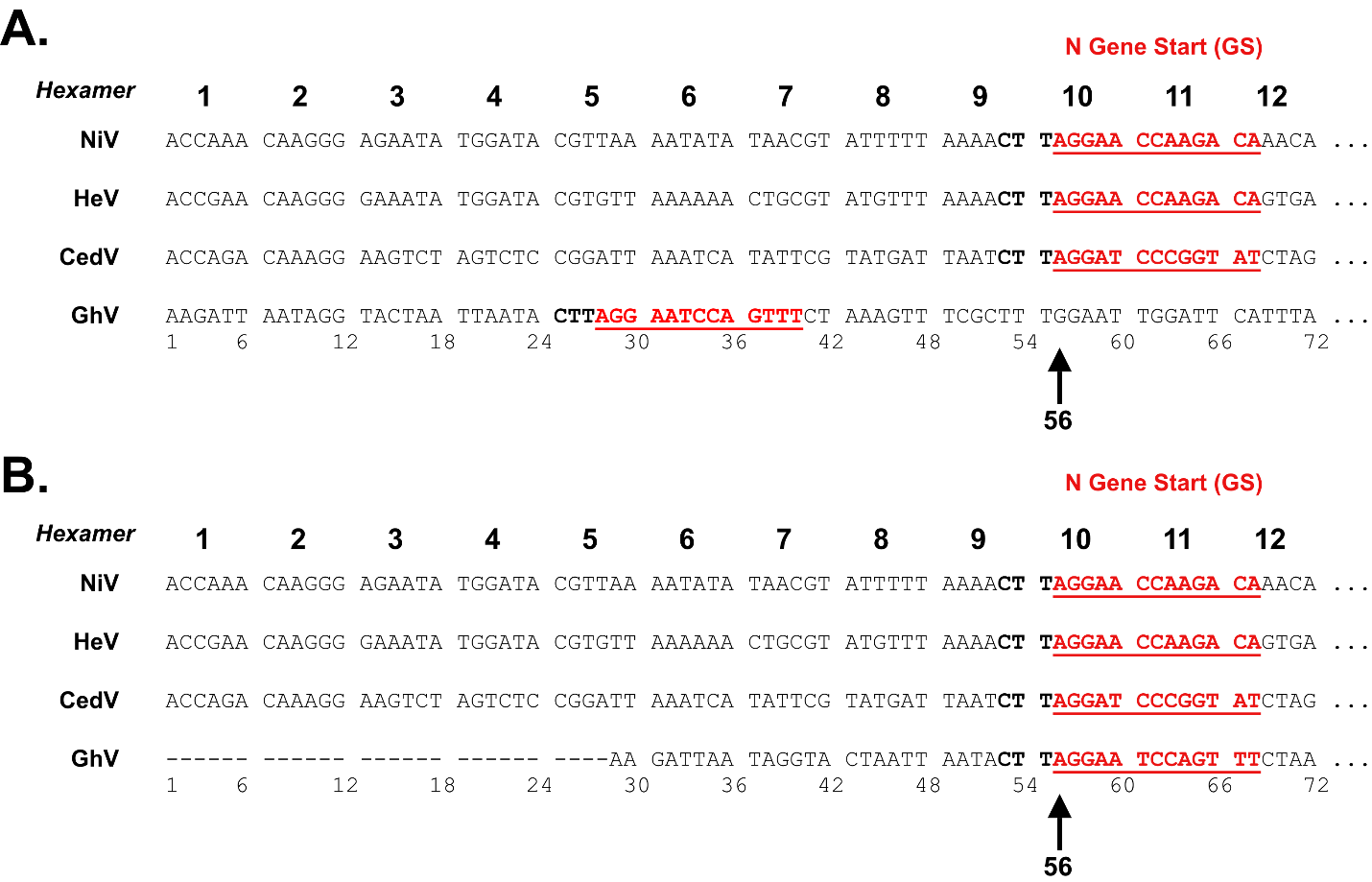


**Supplementary Figure 5. Accounting for unmapped nucleotides in the GhV 3’ Ldr sequence properly phases the GhV N gene start.** (A) Uncorrected sequence alignment of the first 72 nucleotides of the 3’ Ldr sequences of NiV, HeV, and CedV with the reported 3’ Ldr sequence of GhV (all shown as cDNA, 5’ to 3’). (B) Shifting the reported GhV 3’ Ldr sequence by 28 nucleotides effectively positions its N gene start at position 56; hexamer phasing of the N gene start is invariantly conserved among the fully-sequenced bat-borne henipaviruses. For all alignments, the N gene start is colored in red.


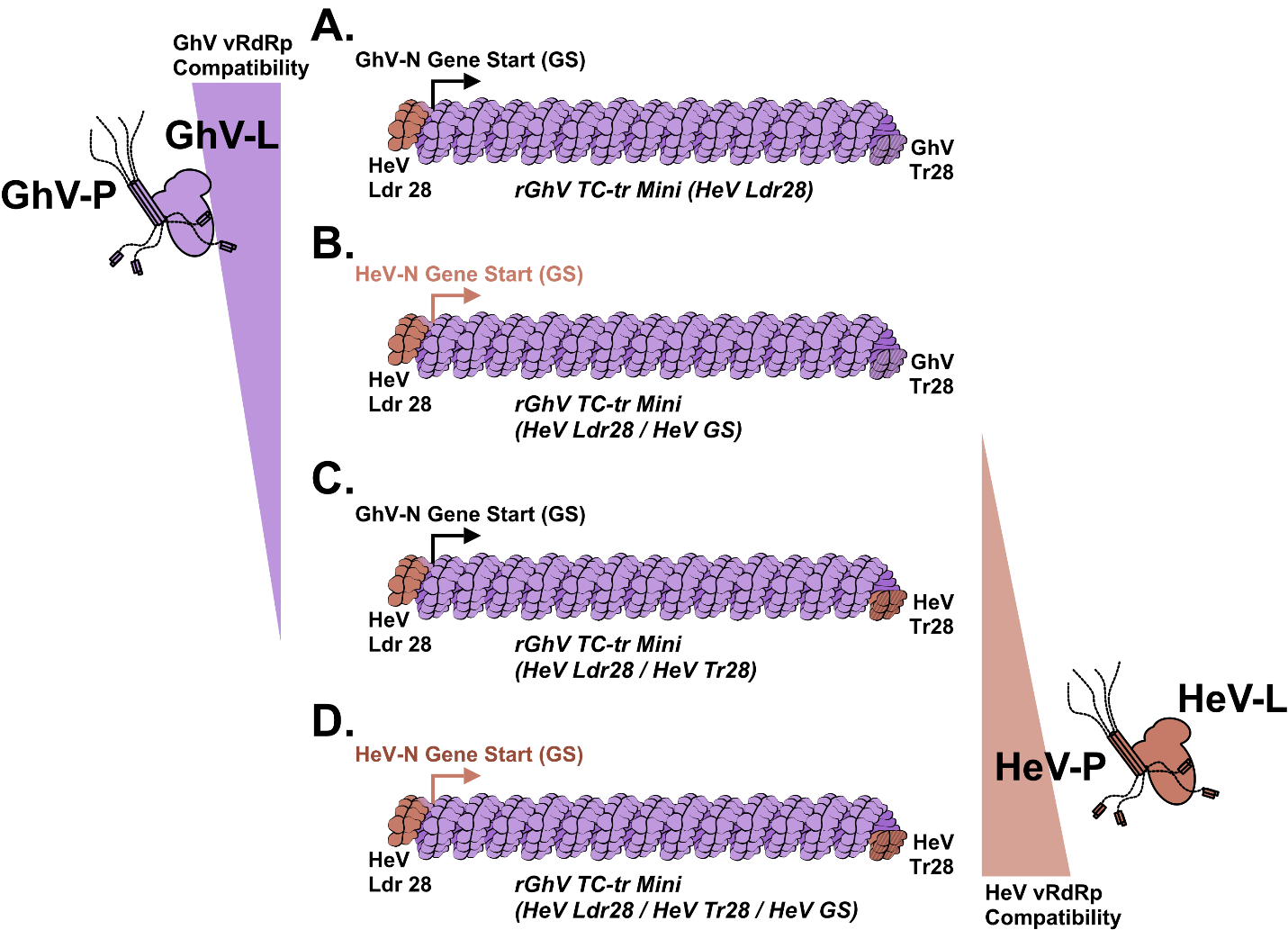


**Supplementary Figure 6. Depiction of rGhV (HeV Ldr28) TC-tr minigenomes with systematic replacement of the GhV-N GS or Tr28 nucleotides with HeV equivalents.** Schematic demonstrates various minigenomes relative to (A) the parental rGhV TC-tr minigenome (HeV Ldr28) construct. (B) Replacement of either the GhV-N GS with the HeV-N GS, or (C) replacement of the terminal 28 nucleotides of the GhV 5’Tr (Tr28) with the HeV equivalent. (D) Combined replacement of both the GhV-N GS and GhV Tr28 sequences with the HeV equivalents. On the left, cartoon signifying which constructs support GhV replicase activity. On the right, carton signifying which constructs support HeV replicase activity.


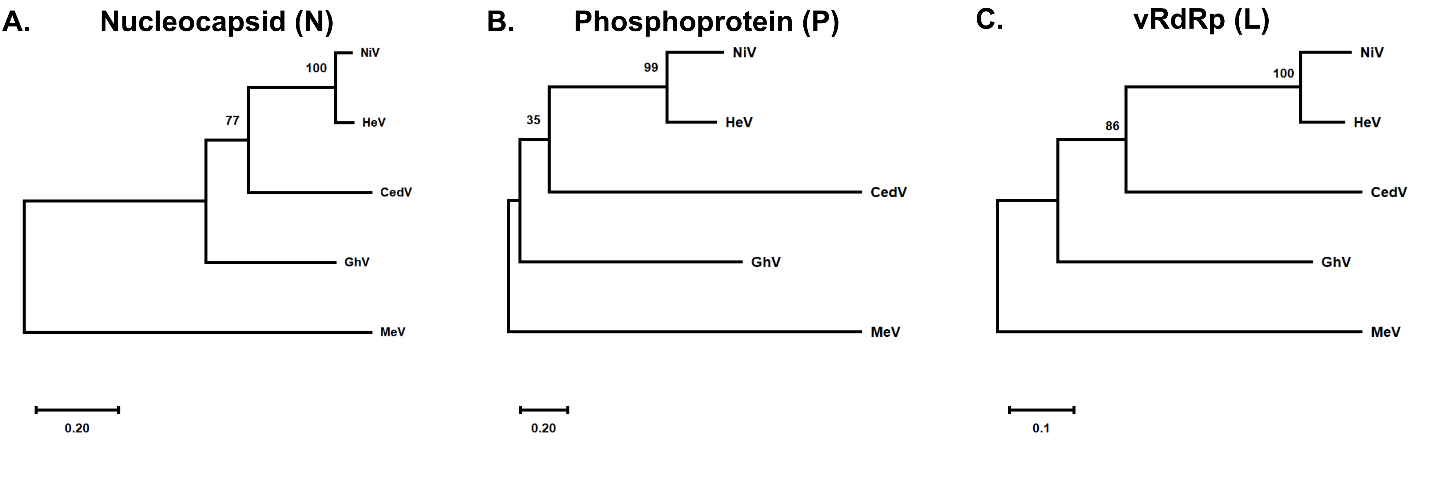


**Supplementary Figure 7. Phylogenetic relatedness of bat-borne HNV replicase proteins.** Phylogenetic trees demonstrating relatedness between the NiV, HeV, CedV, and GhV (A) nucleocapsid, (B) phosphoprotein, and (C) large (vRdRP) proteins. For all analyses, measles virus strain IC323 (MeV) is used as an outgroup. The evolutionary history was inferred by using the Maximum Likelihood method and JTT matrix-based model. The tree with the highest log likelihood is shown. The percentage of trees in which the associated taxa clustered together is shown next to the branches. Initial tree(s) for the heuristic search were obtained automatically by applying Neighbor-Join and BioNJ algorithms to a matrix of pairwise distances estimated using the JTT model, and then selecting the topology with superior log likelihood value. The tree is drawn to scale, with branch lengths measured in the number of substitutions per site. These analyses involved 5 amino acid sequences. Evolutionary analyses were conducted in MEGA X.


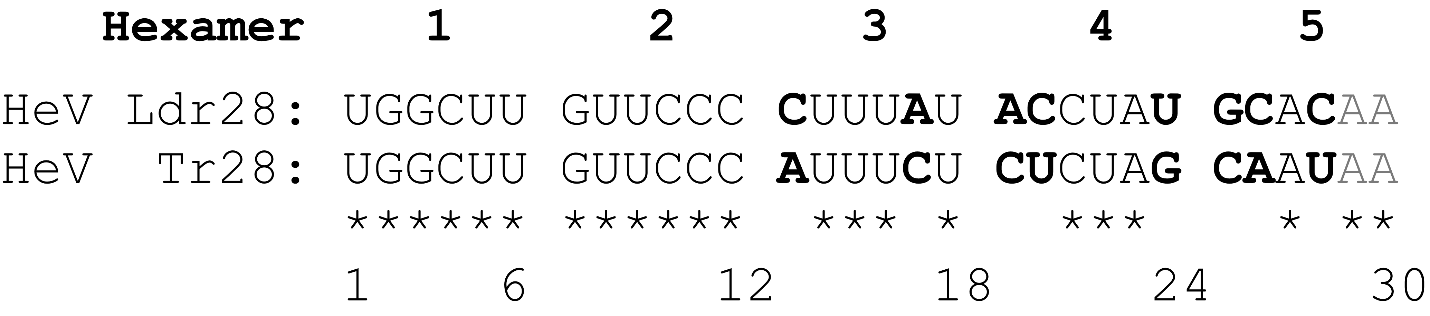


**Supplementary figure 8. Sequence alignment of HeV Ldr28 and Tr28 sequences.** Sequence alignment of the HeV Ldr28 and Tr28 sequences employed in this study. Differences between the two sequences are localized to nucleotide positions 13 and 17 in hexamer 3; positions 19, 20, and 24 in hexamer 4; and positions 25, 26, and 28 in hexamer 5.
